# Evaluation of an Image Analysis Approach to Predicting Primal Cuts and Lean in Light Lamb Carcasses

**DOI:** 10.3390/ani11051368

**Published:** 2021-05-12

**Authors:** Ana Catharina Batista, Virgínia Santos, João Afonso, Cristina Guedes, Jorge Azevedo, Alfredo Teixeira, Severiano Silva

**Affiliations:** 1 Veterinary and Animal Research Center (CECAV), Associate Laboratory of Animal and Veterinary Science (AL4AnimalS), University of Trás-os-Montes e Alto Douro, Quinta de Prados, 5000-801 Vila Real, Portugal; catharina.batista@gmail.com (A.C.B.); vsantos@utad.pt (V.S.); cguedes@utad.pt (C.G.); jazevedo@utad.pt (J.A.); 2 Faculdade de Medicina Veterinária, ULisboa, Avenida da Universidade Técnica, 1300-477 Lisboa, Portugal; jafonso@fmv.ulisboa.pt; 3 Mountain Research Centre (CIMO), Escola Superior Agrária, Instituto Politécnico de Bragança, Campus Sta Apolónia Apt 1172, 5301-855 Bragança, Portugal; teixeira@ipb.pt

**Keywords:** light carcass, cut, video image analysis, prediction

## Abstract

**Simple Summary:**

The traditional way of estimating the carcass composition by complete dissection of muscle, fat and bone is an expensive, time-consuming and inconsistent process. The purpose of this study was to evaluate the accuracy of a simple video image analysis (VIA) system to predict the composition and primal cuts using light lamb carcasses. The six cuts of the carcasses were grouped according to their commercial value: high-value cuts (HVC), medium-value (MVC), low-value (LVC) and all of the cuts (AllC). Results showed the ability of the VIA system to estimate the weight and yield of the groups of carcass joints.

**Abstract:**

Carcass dissection is a more accurate method for determining the composition of a carcass; however, it is expensive and time-consuming. Techniques like VIA are of great interest once they are objective and able to determine carcass contents accurately. This study aims to evaluate the accuracy of a flexible VIA system to determine the weight and yield of the commercial value of carcass cuts of light lamb. Photos from 55 lamb carcasses are taken and a total of 21 VIA measurements are assessed. The half-carcasses are divided into six primal cuts, grouped according to their commercial value: high-value (HVC), medium-value (MVC), low-value (LVC) and all of the cuts (AllC). K-folds cross-validation stepwise regression analyses are used to estimate the weights of the cuts in the groups and their lean meat yields. The models used to estimate the weight of AllC, HVC, MVC and LVC show similar results and a k-fold coefficient of determination (k-fold-R^2^) of 0.99 is achieved for the HVC and AllC predictions. The precision of the weight and yield of the three prediction models varies from low to moderate, with k-fold-R^2^ results between 0.186 and 0.530, *p* < 0.001. The prediction models used to estimate the total lean meat weight are similar and low, with k-fold-R^2^ results between 0.080 and 0.461, *p* < 0.001. The results confirm the ability of the VIA system to estimate the weights of parts and their yields. However, more research is needed on estimating lean meat yield.

## 1. Introduction

In the last decades, studies related to meat characteristics and carcass quality of lambs have been carried out using traditional instrumental methods, such as chemical and physical analyses [1,2]. Physical analyses by complete dissection are the most widely used ways of determining carcass composition. However, the carcass dissection is a time-consuming and expensive process, requiring skilled labor and depreciating the carcass, and is also associated with inconsistency and instability [3,4].

The lack of simple, non-destructive, rapid and reliable methods to assess carcass classification and the characteristics of carcass joints has been one of the barriers to developing quality control systems in the meat industry [5,6]. To overcome these difficulties, several efforts have been employed to develop fast, simple, objective and inexpensive methods of establishing measurements of the carcass and its tissues or cuts [7,8,9].

The research and development of non-destructive, non-invasive technologies have mainly been driven by the need for objective and accurate carcass trait selection, to improve carcass grading or to assess meat quality that satisfies consumer demands, thus add fairness in determining the carcass value and reduce labor requirements for processors [4,10,11]. Video image analysis (VIA) is an example of such technology; it has been widely researched for cattle [3,12,13] and sheep, although to a lesser extent for the latter [7,14]. According to Scholz et al. [6] and Ngo et al. [14], the emphasis on the use of VIA is to imitate visual evaluation, however, in an objective way. The latter authors [14] present a flexible, low-cost and objective image analysis system applied in slaughterhouses, helping determine the cuts and lean prediction weight of lamb carcasses. Usually, the works published on VIA apply to sheep use as far as possible. The carcass spectrum of lambs slaughtered in different production systems typically ranges in weight between 15 and 30 kg [7,8,14]. However, there is a lack of information for light carcasses, and in that regard, this study aims to evaluate the accuracy of a flexible, low-cost VIA system in predicting the weight and yield of lean, commercial cuts from light lamb carcasses.

## 2. Materials and Methods

### 2.1. Animals and Carcasses

Fifty-five lambs of the Portuguese native breed Churra da Terra Quente (CTQ) produced according to the Borrego Terrincho–PDO specifications (Commission regulation EEC no. 1107/96) [15] were used in this work. The animals with 13.5 ± 2.6 kg of live weight were slaughtered in an official slaughterhouse, according to the National and European regulations. After slaughter, the carcasses were refrigerated at 4 °C for 24 h, and the cold carcass weight (CCW) was recorded.

### 2.2. Acquisition of VIA Images and Measurements

Photographic images of the left outer side of each carcass were taken. To acquire the images, each carcass’s left outer side was hung against a dark black background; care was taken to immobilize the carcasses before capturing the side-view image.

**Figure 1:**
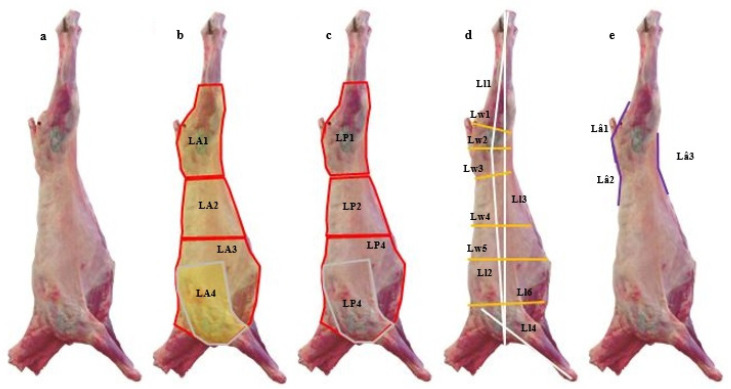
Outer side view of light lamb carcass in Churra da Terra Quente (CTQ) breed (**a**) depicting the descriptors used to collect measures of areas (**b**), perimeters (**c**), lengths and widths (**d**) and angles (**e**). LA1 = area of the leg; LA2 = area of the loin; LA3 = area of the forequarter; LA4 = area of the shoulder; LP1 = perimeter of the leg; LP2 = perimeter of the loin; LP3 = perimeter of the forequarter; LP4 = perimeter of the shoulder; Ll1 = length of the leg; Ll2 = thoracolumbar length; Ll3 = length between the calcaneus and the greater tubercle of humerus; Ll4 = length of the forearm; Lw1 = thinnest width of the leg; Lw2 = largest width of the leg; Lw3 = minimum waist width; Lw4 = maximum waist width; Lw5 = maximum thoracic width; Ll6 = widest part of the chest; Lâ1 = leg angle; Lâ2 = leg angle; Lâ3 = leg angle.

The images were captured using a digital camera (Nikon D3100) with an 8-megapixel sensor. The camera was pre-set as follows: with a manual operation mode, a shutter speed of 1/60s, F/4.5, ISO velocity of 400, without flash and a focal length of 26 mm. Captured images were saved in JPEG format. The entire process was developed under a constant standard artificial light and camera position. The camera was placed at 3 m from the carcasses. Two red dots were projected on a carcass for scale-bar purposes, emitted by two parallel lasers (650 nm wavelength) mounted on a frame with a predetermined distance. The acquired images were transferred to a computer for image analysis. All VIA measurements were performed using the image analysis Fiji software (ImageJ 1.49u) [16]. A total of 21 VIA measurements were recorded for the lateral view images, including areas (4), perimeters (4), angles (3), lengths (4) and widths (6), measured in different regions of the carcass (Figure 1). All measurements were defined as previously [14,17,18].

### 2.3. Carcass Cuts and Composition

The half-carcasses were divided into six cuts: neck, shoulder, breast, rib, loin and leg, as described by Santos et al. [19]. All cuts were dissected into lean, fat and bone according to the methodology proposed by Panea et al. [20] in a room under a controlled environment.

Following the methodology proposed by Rodrigues et al. [21], the cuts were separated into three groups according to their commercial value: high-value group (HVC), which included the leg and loin; medium-value group (MVC) that included the shoulders and ribs; low-value group (LVC) that included the breast, ribs and neck. A fourth group included all of the cuts (AllC). All groups were also calculated as a percentage of CCW.

### 2.4. Models and Statistical Analysis

A descriptive statistical analysis was performed, with the determination of the mean, standard deviation, maximum value, minimum value and coefficient of variation for the weight and lean meat yield for the four cut groups considered and for all VIA measurements. K-folds cross-validation stepwise regression analyses were used to predict the cuts’ weights and percentages and their lean meat (weight and percentage), using CCW plus VIA measurements, or just VIA measurements, as independent variables. The prediction equations for cuts weights and percentages and lean meat weights and percentages were generated separately.

The accuracy of the estimates was based on the k-fold coefficient of determination (k-fold-R2), while the residual standard deviation of the cross-validation (RSDcv) was used to determine the precision of the prediction model. Additionally, as an indicator of the overall predictive ability of the k-fold cross-validation models, the ratio of prediction to deviation (RPD) was also evaluated. The RPD is calculated as the standard deviation (sd) ratio of the reference values to the RSDcv (RPD = SD/RSDcv). An RPD > 2.5 indicates excellent prediction models, 2.0 < RPD < 2.5 indicates good prediction models, 1.8 < RPD < 2.0 indicates good prediction models still allowing quantitative predictions, 1.4 < RPD < 1.8 indicates fair prediction models still useful for assessment and 1.0 < RPD < 1.4 indicates poor prediction models [22]. All statistical procedures were carried out using the JMP software version 14 (SAS Institute, Cary, NC, USA).

## 3. Results

### 3.1. Commercial Dataset Description

Table 1 summarizes the descriptive statistics (mean, standard deviation, minimum, maximum and coefficient of variation) for the lean meat weight and yield of the commercial dataset.

The average weight of the four groups of cuts of commercial value was 1962, 1417, 834 and 4212 g for HVC, MVC, LVC and AllC, respectively. The coefficients of variation for the weight of the cuts in the groups and the lean meat weight varied between 29.09 and 33.27% and between 28.95 and 29.94%, respectively. The coefficient of variation regarding the yield of the cuts and the lean meat yield was smaller, varying from 2.14 to 7.65% and from 4.16 to 6.42%, respectively (Table 1).

The mean, standard deviation, minimum, maximum and coefficient of variation of lateral VIA measurements, as well as the descriptions of linear measurements and carcass dimensions, are presented in Table 2.

In general, area measurements showed greater variation (CV between 19.79 and 25.16%) and the widths showed lower CV variation (between 11.42 and 12.88%; Table 2).

### 3.2. Prediction of Cut Weight and Percentage

Submitting all measurements taken on the carcasses (Figure 1) to the stepwise regression analysis, the final prediction models included 2 to 8 VIA measurements for cut weight, 4 to 5 VIA measurements for cut percentage, 1 to 11 VIA measurements lean meat weight and 2 to 12 VIA measurements for lean meat percentage. Considering all of the final models for cut weights and percentages, and for lean meat weights and percentages, a total of 20 from the initial 21 VIA measurements were used. Recognizing that the use of models with up to 12 VIA measurements would not be practical, the VIA measurements showing lesser contributions to the final models were discarded. Overall, this procedure did not significantly affect the predictive ability nor the precision of the final models and the total number of VIA measurements included in these models was reduced to 12, with a maximum of 5 VIA measurements per model (Table 3, Table 4, Table 5 and Table 6).

Table 3 shows the predictors to estimate the weights of HVC, MVC, LVC and AllC. When CCW was included in the model submitted to the stepwise regression, it was the first independent variable in the final models, representing most of the variation observed (0.959 ≤ k-fold-R^2^ ≤ 0.997, *p* < 0.001). Even with the exclusion of CCW, the final models still explained a large amount of the variation observed (0.836 ≤ k-fold- R^2^ ≤ 0.862, *p* < 0.001).

The ability to predict HVC, MVC, LVC and AllC as percentages of CCW (Table 4) was much smaller, never explaining more than 42.5% (*p* < 0.001) of the variation observed. Even when CCW was included in the analysis, the final models only included this independent variable in the case of MVC% and AllC%.

### 3.3. Prediction of Lean Meat Weight and Percentage

As for cut weights and percentages, the stepwise regression analysis with all of the measurements taken on the carcasses (Figure 1) resulted in final prediction models that included a large number of independent variables—between 1 and 11 VIA measurements for lean mean weight and between 3 and 12 VIA measurements for lean mean percentage. Again, for practical reasons, the VIA measurements showing lesser contributions to the final models were discarded. As observed for cut weights and percentages, overall, this procedure did not significantly affect the predictive ability or the precision of the final models to predict lean meat weights or percentages. Table 5 shows the predictors to estimate the weights of LM_HVC, LM_MVC, LM_LVC and LM_AllC. The pattern was similar to the one observed for cut weights: when CCW was included in the model submitted to the stepwise regression, it was the first independent variable in the final models, explaining almost all of the variation observed (0.956 ≤ k-fold- R^2^ ≤ 0.991, *p* < 0.001). As for cut weights, discarding CCW from the stepwise analysis still provided final models that explained a large amount of the variation observed (0.836 ≤ k-fold- R^2^ ≤ 0.847, *p* < 0.001).

As for HVC, MVC, LVC and AllC, the ability to predict LM_HVC, LM_MVC, LM_LVC and LM_AllC as percentages of CCW (Table 6) was much smaller, compared to the ability to predict the corresponding weights (0.133 ≤ k-fold- R^2^ ≤ 0.438; *p* < 0.001). As for cut percentages, the inclusion of CCW in the stepwise analysis for lean meat percentages resulted in the inclusion of CCW as an independent variable only in the final models for MVC and AllC.

## 4. Discussion

The cuts of the groups separated by commercial value presented in this work follow the specifications of light lambs in the Northern region of Portugal [23] and the southern European countries [24,25,26]. The average weight of the cold carcass was 4.52 kg; that is according to Borrego Terrincho–PDO specifications [23]. The value of the coefficient of variation of the carcass weight reflects the variability of consumer preferences.

Measurements of length, area, width and angle made through VIA systems were previously reported in other studies [14,17,18,27].

The total number of VIA measurements used overall was quite large, even after excluding the poorest predictors, but this was due to the variation in the predictors included in the final models for the different cut groups. A similar variation was recently reported by Gardner et al. [28] in their study using dual-energy X-ray absorptiometry to estimate commercial cut weights. Only the models to estimate cut weights and lean meat weights with CCW excluded from the analysis tended to use the same predictors in the present study. Even in this case, the models for estimating LVC (both in weight and lean meat weight) show some variation.

The present results confirm that carcass weight is the most significant variable in the estimation models for cut weights [14,29]. Brady et al. [29], including carcass weight in their models, had already obtained high accuracies in the prediction of cut weights, explaining 72.2% to 85.8% of the variation of the shoulder, rack, loin and leg cuts. The accuracy of the models now developed is in line with the results of Ngo et al. [14], who, using measurements obtained with a lamb digital grading system, in addition to carcass weight, obtained models that explained 94% to 95% of the variation of primal cut weights. Working with goats, Monteiro et al. [30] also showed an accuracy of above 99% for their predictive model of prime cuts weights, with HCW, by itself, explaining 99% of the variation observed. Rius-Vilarrasa et al. [31] had already reported a high value of models using only VIA measurements to predict cut weights (0.86 < R^2^ < 0.97 for several primal cuts and R^2^ = 0.99 for total primal cuts). Although showing lower predictive value than the models obtained by Rius-Vilarrasa et al. [31], the present results confirmed that VIA measurements, by themselves, are good predictors of cut weights, and their value is not limited to providing complementary information to increase the prediction value of models including CCW as the independent variable.

The poor accuracy of the present models in predicting cut percentages confirms the results obtained by Monteiro et al. [30], with a model based only on one VIA measurement that explained 19.6% of the variation observed in the percentage of primal cuts in goats. However, these results contrast with the high accuracies shown by Brady et al. [29] and Cunha et al. [32], including HCW (and also, in the case of Cunha et al. [32], the longissimus muscle area) in their prediction models. These two studies explained, respectively, 57.9% and 64.1% of the variation of subprimal cut percentages.

Although carcass weight is the most important factor determining the weight of the different cuts, there can be substantial variation in the weight of saleable cuts obtained from carcasses of similar weights, mainly due to variation in fatness, as pointed out by Gardner et al. [28]. The present results indicate that VIA measurements can increase the accuracy of lean meat weight estimates for different cut groups when used as predictors together with CCW and, just by themselves, can provide such estimates with high accuracy.

Concerning lean meat percentage, Normand and Ferrand [33] had already shown little effect of carcass weight, while Stanford et al. [34] showed carcass weight as the main predictor of saleable meat percentage together with VIA measurements. The present results were not clear about this subject, showing CCW as the first predictor to include in models for predicting lean meat percentage in LM_MVC and LM_AllC, but excluding CCW from the models for prediction of the same trait in LM_HVC and LM_LVC. The 36.4% variation explained in the present study for lean meat percentage in LM_AllC, without CCW in the predictive model, is significantly smaller than the 45% variation in lean meat percentage explained by Normand and Ferrand [35] using only VIA predictors. Also, without carcass weight as a predictor, Einarsson et al.’s [27] models explained 60%, 31% and 45% (model 1), and 57%, 30% and 47% (model 2) of the variation observed, respectively, for lean meat percentages of the leg, loin and shoulder. With a model using carcass weight and several VIA measurements, Stanford et al. [34] explained 71% and 62% of the variation observed among lean meat percentages in the leg and shoulder cuts. Including HCW and the longissimus muscle area in their prediction models, Cunha et al. [32] explained 67.8% of the saleable meat yield percentage variation. Except for Einarsson et al.’s [27] models for the loin and shoulder that showed a moderate predictive value of lean meat percentage, these other three studies have shown models with significantly higher predictive values for this trait than the present models.

All models obtained were good predictors (RPD > 2.0) of cut weights and lean mean weights. The ones including CCW, in addition to VIA measurements, were even excellent predictors (RPD > 2.5), as well as the ones without CCW, in the case of MVC and AllC. However, with RPD values between 1.02 and 1.29, the models now obtained showed poor value for predicting cut percentage and lean meat percentage.

## 5. Conclusions

The results of this study confirm and sustain those obtained in other reports concerning the VIA systems’ ability to estimate the weights of different cuts and their lean meat yields. Models combining carcass weight and VIA measurements show increased accuracy with the inclusion of the latter, and models based only on VIA measurements still provide estimates with high accuracy. However, more research is needed to estimate cut percentage and lean meat percentage in light carcass weights of different breeds and with systems producing PDO and Protected Geographical Indication brands.

## Figures and Tables

**Table 1:** Mean, standard deviation (sd), minimum (min), maximum (max) and coefficient of variation (CV) of weight and yield of groups of commercial-value cuts of lamb carcasses.

	Description	Mean	sd	Min	Max	CV (%)
CCW (g)	Cold carcass weight	4523	1324	2162	7622	29.27
Weight (g)						
HVC	High-value cuts	1962	578	946	3442	29.50
MVC	Medium-value cuts	1416	412	700	2298	29.09
LVC	Low-value cuts	834	277	370	1564	33.27
AllC	All cuts	4212	1254	2016	7112	29.78
LM_HVC	Lean meat in HVC	1260	370	574	2244	29.43
LM_MVC	Lean meat in MVC	856	247	410	1408	28.95
LM_LVC	Lean meat in MVC	427	127	204	731	29.94
LM_AllC	Lean meat in all cuts	2543	740	1189	4305	29.11
Percentage of CCW (%)						
HVC	High-value cuts	43.40	1.09	40.00	46.10	2.50
MVC	Medium-value cuts	31.36	1.60	24.90	37.40	5.11
LVC	Low-value cuts	18.28	1.40	15.50	21.60	7.65
AllC	All cuts	93.05	1.99	83.20	98.70	2.14
LM_HVC	Lean meat yield in HVC	27.90	1.17	25.80	31.30	4.21
LM_MVC	Lean meat yield in MVC	18.96	1.22	15.00	22.50	6.42
LM_LVC	Lean meat yield in MVC	9.45	0.57	8.30	10.80	6.08
LM_AllC	Lean meat yield in all cuts	56.30	2.34	51.10	63.80	4.16

CCW = cold carcass weight; HVC = high-value cuts; MVC = medium-value cuts; LVC = low-value cuts; AllC = all cuts; LM_HVC = lean meat in high-value cuts; LM_MVC = lean meat in medium-value cuts; LM_LVC = lean meat in low-value cuts; LM_AllC = lean meat in all cuts.

**Table 2:** Mean, standard deviation (sd), minimum (min), maximum (max) and coefficient of variation (CV) of the video image analysis (VIA) measurements.

Description	Variable	Mean	sd	Min	Max	CV (%)
Area (cm^2^)						
Leg	LA1	185.24	42.41	107.70	271.60	22.89
Loin	LA2	163.06	39.43	97.80	270.60	24.18
Forequarter	LA3	337.77	66.84	204.20	471.30	19.79
Shoulder	LA4	140.50	35.35	83.50	260.70	25.16
Perimeter (cm)						
Leg	LP1	58.64	7.08	44.00	70.70	12.07
Loin	LP2	50.82	7.80	14.00	65.70	15.34
Forequarter	LP3	69.79	10.31	15.30	83.20	14.77
Shoulder	LP4	48.81	8.13	11.40	67.50	16.65
Angle (θ)						
Leg angle 1	Lâ1	142.44	5.77	129.80	153.90	4.05
Leg angle 2	Lâ2	160.43	5.60	149.20	172.80	3.49
Leg angle 3	Lâ3	154.55	9.37	136.00	178.90	6.06
Length (cm)						
Length of the leg	Ll1	30.84	3.36	23.00	38.00	10.89
Thoracolumbar length	Ll2	39.18	4.25	29.70	47.00	10.85
Length between the calcaneus and the greater tubercle of humerus	Ll3	26.89	3.22	18.90	32.40	11.96
Length of the forearm	Ll4	72.72	6.88	55.30	86.40	9.46
Width (cm)						
Thinnest width of leg	Lw1	9.70	1.12	7.20	12.00	11.59
Largest width of the leg	Lw2	10.25	1.29	7.70	13.00	12.58
Minimum waist width	Lw3	9.42	1.19	7.10	12.70	12.58
Maximum waist width	Lw4	13.17	1.70	9.20	16.60	12.88
Maximum thoracic width	Lw5	17.17	2.06	13.10	20.90	11.98
Widest part of the chest	L16	17.40	1.99	13.10	22.70	11.42

**Table 3:** Equations and corresponding coefficient of determination k-fold (k-fold-R^2^), residual standard deviation of the cross-validation (RSDcv) and ratio of prediction to deviation (RPD) for the prediction of cut weight with and without cold carcass weight (CCW) included in the analysis (*n* = 55).

	With CCW Included in the Analysis								With CCW Included in the Analysis							
	HVC		MVC		LVC		AllC		HVC		MVC		LVC		AllC	
Intercept	−16.598		−167.333		−20.561		−149.094		−772.794		−587.231		−560.911		−1811.698	
Independent variables	0.425	CCW	0.289	CCW	0.185	CCW	0.917	CCW	34.901	Ll2	24.69	Ll2	17.178	Ll2	74,348	Ll2
	24.182	Lw2	10.343	Ll4	−14.904	Ll1	1.308	LA2	85.558	L16	62.057	L16	45.212	Lw6	212,244	L16
	−17.278	L16			17.67	L16			−41.504	LP1	−28.220	LP1	−19.362	LP1	−94,902	LP1
	0.661	LA2			1.053	LA2			12.487	LA1	8.702	LA1	4.287	LA1	26,909	LA1
													1.697	LA2		
k-fold- R^2^	0.994		0.977		0.959		0.997		0.849		0.86		0.836		0.862	
RSDcv	46.596		63.914		58.726		75.28		233.533		160.399		118.034		484.759	
RPD	12.42		6.45		4.72		16.66		2.48		2.57		2.35		2.59	

HVC = high-value cuts; MVC = medium-value cuts; LVC = low-value cuts; AllC = all cuts; Ll1 = length of the leg; Ll2 = thoracolumbar length; Ll4 = length of the forearm; Lw2 = largest width of the leg; L16 = widest part of the chest; LA1 = area of the leg; LA2 = area of the loin; LP1 = perimeter of the leg. All models are significant at *p* < 0.001.

**Table 4:** Equations and corresponding coefficient of determination k-fold (k-fold- R^2^), residual standard deviation of the cross-validation (RSDcv) and ratio of prediction to deviation (RPD) for the prediction of cut percentage of the carcass with and without cold carcass weight (CCW) included in the analysis (*n* = 55).

	With CCW Included in the Analysis								Without CCW Included in the Analysis ^#^			
	HVC		MVC		LVC		AllC		MVC		AllC	
Intercept	43.021		27.154		17.772		86.229		30.734		99.551	
Independent variables	0.161	Ll1	−0.001	CCW	−0.314	Ll1	−0.001	CCW	−0.126	Ll2	−0.067	Lâ1
	0.43	Lw2	0.249	Ll4	0.42	L16	0.696	Lw3	0.206	Ll4	0.019	LA2
	−0.516	L16			0.018	LA2	0.024	LA2				
k-fold- R^2^	0.219		0.124		0.425		0.214		0.077		0.16	
RSDcv	0.977		1.527		1.089		1.816		1.568		1.858	
RPD	1.12		1.05		1.29		1.1		1.02		1.07	

HVC = high-value cuts; MVC = medium-value cuts; LVC = low-value cuts; AllC = all cuts; Ll1 = length of the leg; Ll2 = thoracolumbar length; Ll4 = length of the forearm; Lw2 = largest width of the leg; L16 = widest part of the chest; LA2 = area of the loin; Lâ1 = leg angle. All models are significant at *p* < 0.001. ^#^ For HVC and LVC, the final prediction model was similar to the one obtained including ‘with CCW’ in the analysis.

**Table 5:** Equations and corresponding coefficient of determination k-fold (k-fold- R^2^), residual standard deviation of the cross-validation (RSDcv) and ratio of prediction to deviation (RPD) for the prediction of lean meat weight with and without cold carcass weight (CCW) included in the analysis (*n* = 55).

	With CCW Included in the Analysis								Without CCW Included in the Analysis							
	LM_HVC		LM_MVC		LM_LVC		LM_AllC		LM_HVC		LM_MVC		LM_LVC		LM_ALLCuts	
Intercept	−77.604		−201.327		−24.377		−176.522		−873.782		−575.884		−13.793		−1813.733	
Independent variables	0.254	CCW	0.175	CCW	0.086	CCW	0.498	CCW	69.959	L16	47.266	L16	1.881	LA1	148.297	L16
	12.243	Ll1	−8.805	Ll2	0.397	LA2	19.008	Ll1	4.95	LA1	3.292	LA1	0.757	LA2	9.595	LA1
	−22.046	L16	11.069	Ll4			−11.557	Ll2					−4.425	LP4		
	1.040	LA1	5.728	Ll3			1.798	LA1					1.317	LA4		
			−0.625	LA2												
k-fold- R^2^	0.989		0.976		0.956		0.991		0.836		0.838		0.843		0.847	
RSDcv	40.001		40.109		27.438		72.954		153.157		101.537		52.696		295.271	
RPD	9.27		6.18		4.66		10.15		2.42		2.44		2.43		2.51	

LM_HVC = lean meat in high-value cuts; LM_MVC = lean meat in medium-value cuts; LM_LVC = lean meat in low-value cuts; LM_AllC = lean meat in all cuts; Ll1 = length of the leg; Ll2 = thoracolumbar length; Ll3 = length between the calcaneus and the greater tubercle of humerus; Ll4 = length of the forearm; L16 = widest part of the chest; LA1 = area of the leg; LA2 = area of the loin; LP4 = perimeter of the shoulder; LA4 = area of the shoulder. All models are significant at *p* < 0.001.

**Table 6:** Equations and corresponding coefficient of determination k-fold (k-fold- R^2^), residual standard deviation of the cross-validation (RSDcv) and ratio of prediction to deviation (RPD) for the prediction of lean meat percentage with and without cold carcass weight (CCW) included in the analysis (*n* = 55).

	With CCW Included in the Analysis								Without CCW Included in the Analysis ^#^			
	LM_HVC		LM_MVC		LM_LVC		LM_AllC		LM_MVC		LM_AllC	
Intercept	26.829		16.257		10.423		51.198		18.062		37.971	
Independent variables	0.325	Ll1	−0.001	CCW	−0.048	Ll2	−0.002	CCW	−0.148	Ll2	0.488	Ll1
	−0.174	Ll2	−0.101	Ll2	0.006	LA2	0.495	Ll1	0.311	Ll4	−0.390	Ll2
	−0.487	L16	0.266	Ll4			−0.274	Ll2	−0.010	LA2	0.130	Lâ1
	0.107	LP1	0.017	LA1			0.043	LA1				
k-fold- R^2^	0.433		0.357		0.133		0.438		0.287		0.364	
RSDcv	0.919		1.013		0.547		1.827		1.056		1.923	
RPD	1.27		1.20		1.04		1.28		1.16		1.22	

LM_HVC = lean meat in high-value cuts; LM_MVC = lean meat in medium-value cuts; LM_LVC = lean meat in low-value cuts; LM_AllC = lean meat in all cuts; Ll1 = length of the leg; Ll2 = thoracolumbar length; Ll4 = length of the forearm; L16 = widest part of the chest; LA1 = area of the leg; LA2 = area of the loin; Lâ1 = leg angle; LP1 = perimeter of the leg. All models are significant at *p* < 0.001. ^#^ For LM_HVC and LM_LVC, the final prediction model was similar to the one obtained including ‘with CCW’ in the analysis.

## Data Availability

The data presented in this study are available on request from the corresponding author.
